# PSMP Is Discriminative for Chronic Active Antibody-Mediated Rejection and Associate With Intimal Arteritis in Kidney Transplantation

**DOI:** 10.3389/fimmu.2021.661911

**Published:** 2021-04-09

**Authors:** Panpan Zhan, Haizheng Li, Mingzhe Han, Zhen Wang, Jie Zhao, Jinpeng Tu, Xiaofeng Shi, Yingxin Fu

**Affiliations:** ^1^ Department of Kidney Transplantation, Tianjin First Central Hospital, School of Medicine, Nankai University, Tianjin, China; ^2^ Department of Kidney Transplantation and Kidney Transplantation Research Laboratory, Tianjin First Central Hospital, Tianjin, China; ^3^ Key Laboratory of Transplantation, Chinese Academy of Medical Sciences, Tianjin, China; ^4^ First Central Clinical College of Tianjin Medical University, Tianjin, China; ^5^ Institute of Hematology & Blood Diseases Hospital, State Key Laboratory of Experimental Hematology, National Clinical Research Center for Blood Diseases, Chinese Academy of Medical Sciences & Peking Union Medical College, Tianjin, China

**Keywords:** kidney transplantation, chronic active antibody-mediated rejection, PC3-secreted microprotein, macrophages, intimal arteritis

## Abstract

Chronic active antibody-mediated rejection (CAAMR) is an intermediate process that occurs during the development of chronic antibody-mediated rejection (CAMR), which is a key problem associated with the long-term kidney grafts survival. This study investigated the role played by PC3-secreted microprotein (PSMP) in the progression of CAAMR and CAMR. We showed that CAAMR and CAMR patients’ allografts dysfunction with declined survival rate, which suggested that earlier diagnosis and treatment of CAAMR might be important to prevent irreversible chronic injury of CAMR progression. We found PSMP was an important factor in the development of chronic antibody-mediated rejection. The PSMP expression increased significantly in CAAMR biopsy samples but not in CAMR and control patients, which distinguished CAAMR patients from CAMR and non-rejection patients. Moreover, our results showed that infiltration of CD68^+^ macrophages in CAAMR increased, and the correlation between CD68^+^ macrophages and PSMP expression in CAAMR patients was significant. Additionally, our data also revealed that intimal arteritis (v-lesion) accompanied by increased macrophage infiltration might have contributed to more graft loss in CAAMR, and PSMP expression was significantly associated with the v-lesion score. These results indicated that PSMP played an important role in the recruitment of macrophages and promote intimal arteritis inducing allograft lost in CAAMR progression. In future study PSMP could be a potential histopathological diagnostic biomarker and treatment target for CAAMR in kidney transplantation.

## Introduction

Chronic active antibody-mediated rejection (CAAMR) is an intermediate process that occurs during the development of chronic antibody-mediated rejection (CAMR), which has been recognized recently. CAAMR leads to the gradual loss of allograft, becoming an obstacle to the long-term survival of renal allografts (1). Significant improvements in short-term renal graft survival have been achieved in recent decades due to the continuous updating of immunosuppressive agents, such as calcineurin inhibitors, which greatly reduce the occurrence of T cell-mediated rejection (TCMR) (2). However, long-term renal allograft loss caused by CAAMR has no significant improvements without effective therapeutic drugs (3, 4). Although the diagnostic criteria for CAAMR were defined in the revised Banff 2017 criteria (5, 6), many morphological lesions associated with CAAMR and CAMR appear similar and it’s difficult to distinguish this two phases in clinic clearly. Moreover, it’s lacking specific molecular pathological biomarkers available for expressing the intermediate injury from CAAMR progress to CAMR (7).

Increasing attention has been paid to macrophage graft infiltration in the immunopathological characteristics of chronic allotransplantation rejection (8). Macrophages are a type of innate immune cell that participate in adaptive immunity through antigen presentation, co-stimulation, tissue repair, and the production of pro-inflammatory cytokines. Macrophages may be recruited to the rejection site, augmenting the immune response and promoting the renal glomeruli and tubules injury. The persistent inflammation mediated by macrophages may lead to fibrosis and chronic rejection in renal allograft (9). Macrophage infiltration one year after transplantation has been demonstrated to be associated with graft dysfunction and fibrosis (10). The evidence shows that CD68^+^CD163^+^ macrophages tend to increase in CAAMR compared with acute antibody-mediated rejection (ABMR) and TCMR (11, 12), which may promote the chronic progressive injury. Macrophages found in renal allografts can include resident macrophages from donor tissues and blood-derived macrophages from recipients. However, recent studies have shown that macrophages associated with chronic rejection are primarily derived from renal transplant recipients (13). Peripheral circulating macrophages can be recruited into grafts by a variety of chemokines. PC3-secreted microprotein (PSMP) is a newly identified chemokine found in the PC3 cell line and malignant prostate tumors (13). PSMP has a similar affinity for C-C motif chemokine receptor 2 (CCR2) as that of C-C motif chemokine ligand 2 (CCL2). PSMP can recruit monocytes from the peripheral blood through interactions with CCR2, mediating macrophage infiltration in tissue. Recent studies have shown that PSMP plays an important role in liver fibrosis in humans and mice. PSMP promotes the infiltration and polarization of inflammatory macrophages which cause liver fibrosis through interactions with CCR2. The administration of a PSMP neutralizing antibody can significantly improve liver fibrosis in mice (14), indicating that PSMP plays a key role in the pathogenesis of inflammation-related diseases.

In this study, we explored the roles played by PSMP in the progression of CAAMR and CAMR. We showed that the expression of PSMP was significantly increased in CAAMR patients but not in CAMR patients, suggesting that PSMP represent a significant discriminative marker between CAAMR and CAMR patients. A significant correlation was found between PSMP expression and CD68^+^ macrophages infiltration in CAAMR patients, and PSMP expression levels were significantly associated with intimal arteritis, which indicated that PSMP might play an important role in CAAMR.

## Materials and Methods

### Study Population and Samples

**Figure 1:**
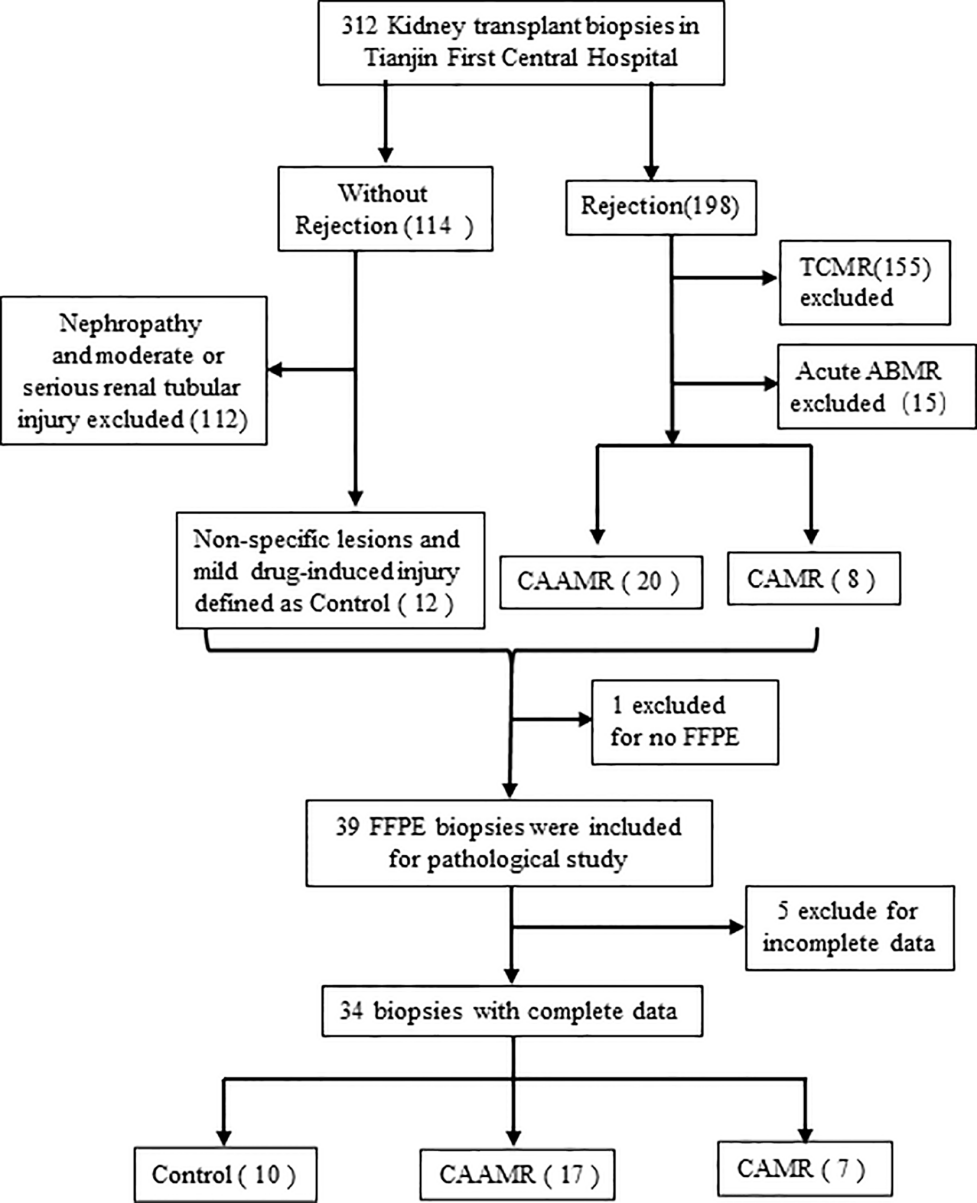
Flow chart of biopsies inclusion and exclusions for the study. We collected 312 kidney biopsy and selected 198 biopsies with an original diagnose of rejection and 114 subjects without rejection. 20 biopsies were redefined as CAAMR and 8 biopsies were defined as CAMR by a central pathologist. In 114 subjects without rejection, 12 patients diagnosed with non-specific lesions or mild drug-induced injuries were defined as Control. We excluded 1 subject with incomplete FFPE slides and 5 subjects with incomplete central pathology data. TCMR, T cell-mediated rejection; ABMR, antibody-mediated rejection; FFPE, formalin-fixed paraffin-embedded.

We retrospective studied 312 patients who underwent kidney biopsy between July 2017 and October 2020 in Tianjin First Central Hospital. We selected 198 biopsies with an original diagnose of rejection, 20 biopsies were re-evaluated and defined as CAAMR, 8 biopsies were defined as CAMR according to the 2017 revised Banff criteria (15). In 114 subjects without rejection, 12 patients diagnosed with non-specific lesions or mild drug-induced injuries were defined as Control. We excluded 1 subject with incomplete formalin-fixed paraffin-embedded (FFPE) slides and 5 subjects with incomplete central pathology data, resulting in the inclusion of 34 subjects (10 Control, 17 CAAMR, 7 CAMR) in the final analysis (**Figure 1**). The sample size was set to 6 for feasibility reasons in each group. Assuming an effect size of about 1.6 and provide 80% power using Tukey’s test, two-sided significance level of 0.05. Urine and blood samples were collected at the time of the biopsy. All patients underwent ABO-compatible renal transplantations, and biopsies were obtained from all patients for clinical surveillance due to elevated creatinine or proteinuria. The collection of human samples was approved by the Ethics Committee of Tianjin First Central Hospital and was performed according to the Declaration of Helsinki guidelines.

The diagnostic criteria of CAAMR follow the 2017 revised Banff criteria as follows (1): At least 1 AMR chronicity histologic features: – Banff Lesion Score cg > 0. – 7 or more layers in 1 cortical peritubular capillary (ptc) and 5 or more in 2 additional capillaries. – Arterial intimal fibrosis of new onset (2). At least 1 criterion of antibody interaction with tissue. –At least moderate MVI (g + ptc > 1) in the absence of glomerulonephritis. If suspicious (Borderline) for acute T cell-mediated rejection (TCMR), acute TCMR, or infection is present, Banff Lesion Score g>1 is required (3). At least 1 criterion of DSA or equivalents: – DSA positive (anti-HLA or other specificity). – Banff Lesion Score C4d > 1 (IF on fresh frozen tissue) or C4d > 0 (IHC on FPE tissue).

### Immunohistochemistry

We used a PSMP polyclonal antibody [3D5, purified and provided by the Institute of Hematology and Blood Diseases Hospital, Chinese Academy of Medical Science, Beijing, China (16)] to detect the expression of PSMP in renal biopsies. In brief, the paraffin sections were dewaxed by baking for 90 min at 65°C and deparaffinized in xylene solutions and various alcohol concentrations (100%, 95%, and 75%). The sections were placed in a 3% hydrogen dioxide solution for 8 min and boiled with citrate buffer solution for 15 min. The sections were then blocked with serum for 0.5 h. The PSMP polyclonal antibody, anti-CD68 (1:100; ab125212, Abcam Inc., Cambridge, MA, USA), anti-CD163 (1:50, ZM-0428, Zhongshan Bridge, Beijing, China) were used as primary antibodies, and incubation was performed at 4°C overnight. A biotin-conjugated goat anti-mouse/rabbit IgG antibody (1:100, Zhongshan Bridge, Beijing, China) was used as the secondary antibody, and incubation was performed at 37°C for 30 min. The tissues were colored with diaminobenzidine (DAB) solution (Vector Laboratories, Inc., Burlingame, USA). A semi-quantitative assessment was conducted by Image-Pro Plus software (Image-Pro Plus 6.0, USA), the average positive cells number in at least 5 high-power field (HPF, 40×) or mean integrated optic density (IOD) was calculated.

### RNA Isolation and Real-Time Polymerase Chain Reaction (RT-PCR)

RNA was isolated from biopsies using the Qiagen RNA microextraction kit (Qiagen, Valencia, CA), according to the manufacturer’s instructions. RNA was reverse transcribed into cDNA using the cDNA synthesis kit (Invitrogen, Carlsbad, CA, USA) according to the manufacturer’s instructions. The SYBR Premix RT-qPCR kits (Bio-Rad Laboratories, Hercules, CA) and Applied Biosystems GenAmp 7700 sequence detection system (Applied Biosystems, CA, USA) were used for the quantitative detection of mRNA levels. Glyceraldehyde 3-phosphate dehydrogenase (*GAPDH*) was employed as the normalization control. The levels of relative gene expression were computed using the 2^−ΔΔCt^ approach. The following primers were used: *PSMP*, fw: 5’-CTGTGACACGGCTCAGCATC-3’, rev: 5’-ATGGGCAAGCCTTTAGCTGG-3’; *GAPDH*, fw: 5’-AGGTCGGTGTGAACGGATTTG-3’, rev: 5’-TGTAGACCATGTAGTTGAGGTCA-3’.

### Cytokine Bead Assay

Microspheres (A37304, Life Technologies) coated with anti-rabbit polyclonal antibody were used as capture antibodies to detect the expression of PSMP in blood and urine as described (17). Gradient concentrations of PSMP monoclonal antibodies were used as detection antibodies, and phycoerythrin (PE)-labeled rabbit anti-mouse antibodies were used as secondary antibodies. The fluorescence intensity of microspheres was detected by flow cytometry, and analysis by logarithms. Finally, linear fitting was performed to obtain a standard curve, and the PSMP level was calculated in blood and urine samples.

### Statistical Analysis

All data are processed by R3.6.2 statistical software. Normally distributed data are expressed as mean ± SD, comparison between groups using one-way analysis of variance (ANOVA) or 2-tailed Student’s t-test. The nonnormally distributed data are expressed as median (interquartile range), comparison between groups using nonparametric Kruskal–Wallis test or nonparametric Mann–Whitney U test. Correlations between PSMP expression and other variables were analyzed by Pearson’s correlation coefficient. Receiver operator characteristic (ROC) curve analysis was used to calculate the cut-off value for PSMP protein levels in renal biopsies and to assess the diagnostic ability of PSMP in CAAMR patients. Graft survival was analyzed by Kapla-Meier analysis, and survival curve was compared by Log-rank test. A value of p ≤ 0.05 was considered significant. ∗p ≤ 0.05, ∗∗p ≤ 0.01, and ∗∗∗p ≤ 0.001.

## Results

### Patients’ Baseline Characteristics

A total of 34 patients who underwent biopsy were selected and divided into three groups: Control (10/34), CAAMR (17/34), and CAMR (7/34). A comparison of the clinical characteristics among the three groups was summarized in **Table 1**. The CAAMR and CAMR groups presented positive anti‐human leukocyte antigen (HLA) donor-specific antibodies (DSA) at the time of biopsy compared to Control group (p < 0.05), and 14 of 17 CAAMR patients were positive for anti‐HLA class II antibody, as were 4 of 7 in CAMR patients. No significant difference was observed among the three groups for baseline characteristics including age, gender, retransplantation, infections, diabetic nephropathy, time posttransplant to biopsy, HLA mismatch, PRA pre-transplant or immunosuppressive regimen (p > 0.05).

**Table 1:** Patients Baseline Characteristics of kidney transplant patients.

Characteristics	Normal (n=10)	CAAMR (n=17)	CAMR (n=7)	p value
Age, year-mean ± SD	36.6 ± 14.49	44.78 ± 15.46	41.47 ± 14.09	0.68
Male sex- n (%)	8 (80%)	14(82.35%)	6 (85.71%)	0.99
Donor (relative)-n (%)	0 (0%)	1 (5.88%)	1 (14.29%)	0.52
Retransplantation-n (%)	0 (0%)	0 (0%)	1 (14.29%)	0.18
Time posttransplant to biopsy, days- Median (Q1-Q3)	606(545.75- 892.5)	606(614.75- 2051.5)	1932(735- 2099.5)	0.29
Biopsy times(n)	1.2 ± 0.4	1.35 ± 0.58	1.43 ± 0.49	0.66
Infections-n (%)	3 (30%)	5 (29.41%)	2 (28.57%)	0.99
Diabetic nephropathy, (%)	2 (20%)	6 (35.29%)	0 (0%)	0.3
Diabetes after transplantation-n (%)	1 (10%)	1 (5.88%)	1 (14.29%)	0.83
HLA mismatch-mean ± SD				
HLA A/B mismatch	3.33 ± 0.94	2.91 ± 0.95	2.71 ± 0.88	0.44
HLA DR mismatch	1.66 ± 0.47	1.75 ± 0.43	1.43 ± 0.73	0.49
cPRA(%)	66.2 ± 11.5	64.9 ± 13.6	64.6 ± 17.5	0.97
PRA pretransplantation	2 (20%)	2 (11.76%)	1 (14.29%)	0.88
Anti-HLA DSA at the time of biopsy-n (%)	0 (0%)	14(82.35%)	5 (71.43%)	0.03
Class I	0 (0%)	0 (0%)	1 (14.29%)	
Class II	0 (0%)	12 (70.59%)	4 (57.14%)	
Class I+II	0 (0%)	2 (11.76%)	0 (0%)	
Immunosuppressive therapy- n (%)				
CNI				0.71
FK-n (%)	8 (80%)	10 (58.82%)	6 (85.71%)	
CsA-n (%)	2 (20%)	6 (35.29%)	1(14.29%)	
Other-n (%)	0 (0%)	1(5.88%)	0 (0%)	
MMF				0.39
Mythology-n (%)	5 (50%)	9 (52.94%)	6 (85.71%)	
Myfortic-n (%)	5 (50%)	5 (29.41%)	0 (0%)	
Other-n (%)	0 (0%)	3 (17.65%)	1 (14.29%)	
Pred				
Prednisone-n (%)	10 (100%)	18 (100%)	7 (100%)	1
Allograft loss-n (%)	0	5 (29.41%)	3 (42.86%)	0.19

HLA, histocompatibility leukocyte antigen; cPRA, calculated panel reactive antibody; PRA, panel reactive antibody; DSA, donor-specific antibodies; CNI, Calcineurin inhibitor; FK, tacrolimus; CSA, cyclosporin A; MMF, mycophenolate mofetil; Pred, prednisone; CAAMR, Chronic active antibody-mediated rejection; CAMR, chronic antibody-mediated rejection.

### Declined Graft Function and Survival Rate in CAAMR and CAMR Patients

**Figure 2:**
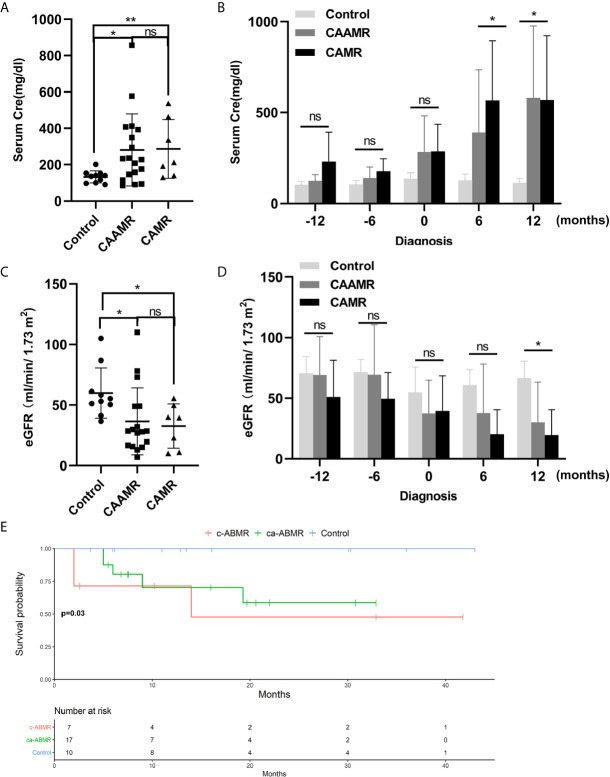
Declined graft function and survival rate in CAAMR and CAMR patients. The serum creatinine **(A, B)** and eGFR **(C, D)** in all patients (Control, CAAMR, CAMR) were collected and analyzed at the time of diagnosis of rejection and from one year before biopsy to one year after biopsy. Kaplan-Meier survival analysis was used to compare the biopsy time to allograft failure between the three groups with the log-rank test **(E)**. Cre, creatinine; eGFR, estimated glomerular filtration rate. *p ≤ 0.05, **p ≤ 0.01. ns, no significance.

We evaluated the changes of serum creatinine and estimated glomerular filtration rate (eGFR) in CAAMR and CAMR patients compared with Control patients without rejection from one year before the diagnosis of rejection to one year after diagnosis, which reflecting renal function during chronic rejection progression. The serum creatinine level was significantly higher and increased after diagnosis in CAAMR and CAMR patients compared to control patients (**Figures 2A, B** **)**, while eGFR (**Figures 2C, D** **)** was opposite to that of creatinine, showed a declined renal graft function. Kaplan-Meier survival analysis showed that both the CAAMR and CAMR groups have lower survival rates than the non-rejection group (p < 0.05; **Figure 2E**).

### Patients’ Pathological Features

**Figure 3:**
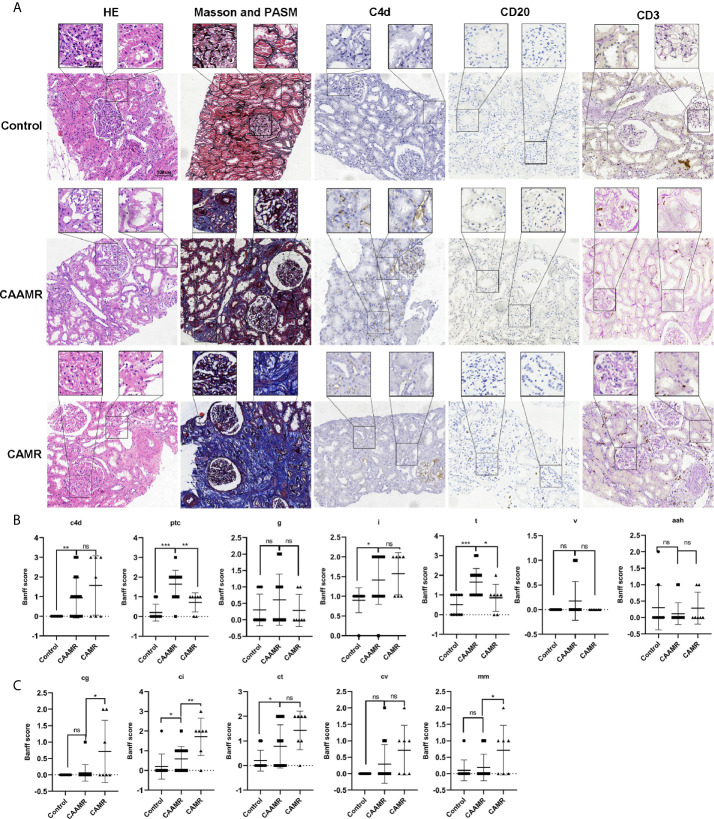
Patients’ pathological features. Patients pathological data included HE, Masson and PASM, C4d, CD20, CD3 staining **(A)** was collected and shown. Banff scores included C4d, ptc, g, i,t, v, aah **(B)** and cg, ci, ct, cv, mm **(C)** were collected and compared among three groups. HE, hematoxylin and eosin; PASM, periodic acid-silver metheramine. *p ≤ 0.05, **p ≤ 0.01, and ***p ≤ 0.001. ns, no significance.

We collected the clinicopathological data for all patients, including hematoxylin and eosin (HE), Masson’s trichrome and periodic acid-silver metheramine (PASM), CD20, C4d, CD3, and CD20 staining as shown in **Figure 3A**. We analyzed the differences in Banff scores among the three groups. The CAAMR group showed higher acute Banff scores include peritubular capillary (PTC), Glomerulitis (g), tubulitis (t), intimal arteritis(v) than those in CAMR and Control groups (**Figure 3B**). The CAAMR and CAMR groups showed significantly higher C4d score and chronic scores include interstitial fibrosis (ci), glomerular double contour (cg) and tubular atrophy (ct) than those in the Control group, CAMR patients revealing more distinct chronic characteristics (**Figure 3C** **).**

### PSMP Expression in Biopsy Tissue Was a Biomarker for the Diagnosis of CAAMR

**Figure 4:**
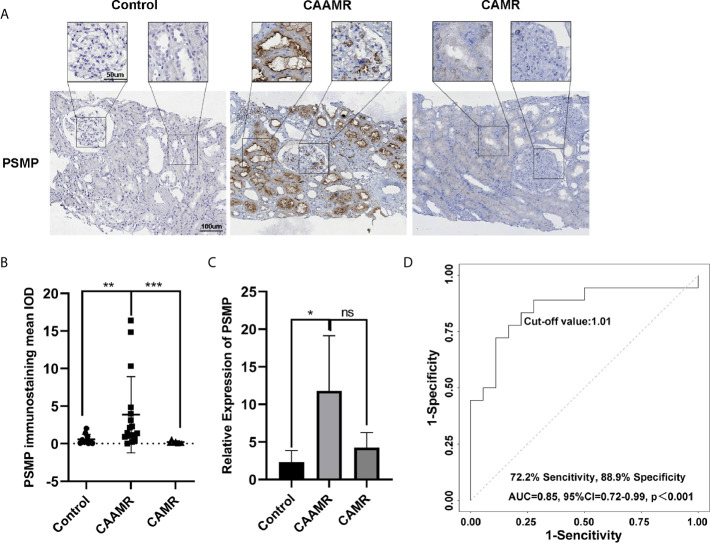
PSMP expression in biopsy tissue may be a biomarker for the diagnosis of CAAMR. The expression of PSMP protein was detected by immunohistochemistry **(A)** and quantified by Image-pro plus software **(B)**. PSMP mRNA level was analyzed by RT-PCR in biopsy tissues **(C)**. Receiver operating characteristic (ROC) curves were used to evaluate the value of PSMP protein expression in the prediction of CAAMR from Control and CAMR patients **(D)** in graft biopsies. *p ≤ 0.05, **p ≤ 0.01, and ***p ≤ 0.001. ns, no significance.

In this study, we focused on PSMP because it is a chemokine with increased expression at the site of the inflammatory injury. The immunohistochemistry analysis showed that PSMP protein was strongly expressed in the CAAMR group compared with the Control and CAMR groups (p < 0.01, **Figures 4A**, **B**). The PSMP mRNA expression levels in biopsy tissue were analyzed for all three groups, which showed that PSMP mRNA was detected at significantly higher levels in the biopsy tissues of CAAMR patients (five cases) compared with those from the Control group (3 cases, p < 0.05). No significant difference in mRNA levels was observed between the CAAMR and CAMR (3 cases) samples, which might because of the low sample size (**Figure 4C**). The ROC curve analysis showed that the PSMP protein expression level in biopsy tissues was a good discriminator for distinguishing CAAMR patients from Control and CAMR patients, with an area under the ROC curve (AUC) of 0.85 (95% confidence interval, 0.72 to 0.99), a specificity of 88.9%, and a sensitivity of 72.2% (p < 0.001, **Figure 4D**). We performed Kaplan-Meier survival analysis at the cut-off value of PSMP protein in biopsy, which revealed no significant differences in graft survival between high- and low-PSMP expression groups among CAAMR patients (**Figure S1A**).

### PSMP Expression Was Related to Macrophage Infiltration in CAAMR Patients

**Figure 5:**
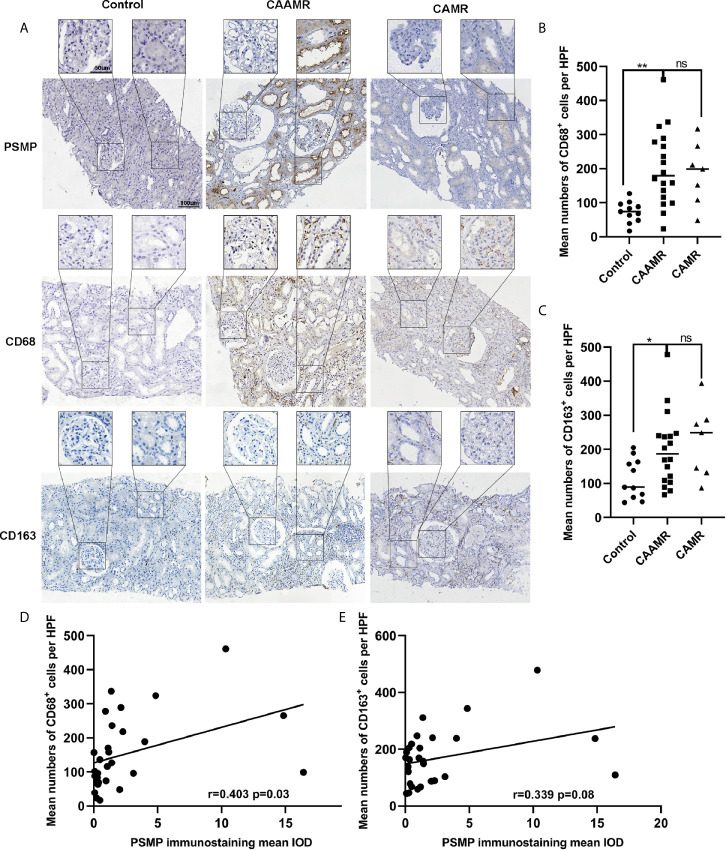
The relationship between PSMP expression and macrophage infiltration in CAAMR patients. The CD68^+^ and CD163^+^ cells infiltration was detected by immunohistochemistry **(A)** and quantified as average number of positive cells per HFP (40×) by Image-pro plus software **(B, C)**. Correlations between PSMP expression and CD68^+^ **(D)** and CD163^+^ **(E)** cells infiltration were analyzed by Pearson’s correlation coefficient. HPF, high-power field. *p ≤ 0.05, **p ≤ 0.01. ns, no significance.

Recently, macrophages were suggested to play a more immediate role in allograft rejection. CD68 and CD163 immunohistochemistry staining were performed in biopsies for the quantitative analysis of macrophage infiltration, which showed a higher number of CD68^+^ cells per HPF in CAAMR and CAMR patients than in Control patients (p < 0.01, **Figures 5A, B** **)**. The CD163^+^ cells number was higher in CAAMR patients compared with control patients (p < 0.05) but lower than CAMR patients (**Figure 5C**). Because PSMP expression levels were higher in CAAMR patients, we used Spearman’s correlation coefficient to analyze the correlation between PSMP expression and CD68^+^ or CD163^+^ infiltrating cells in the CAAMR and Control groups, which revealed a significant correlation between PSMP expression and the number of infiltrating CD68^+^ cells (p < 0.05, **Figure 5C**), but no significant correlation between PSMP expression and the number of CD163^+^ cells (**Figure 5E**).

### PSMP Expression Was Related to V-Lesion in CAAMR Patients

**Figure 6:**
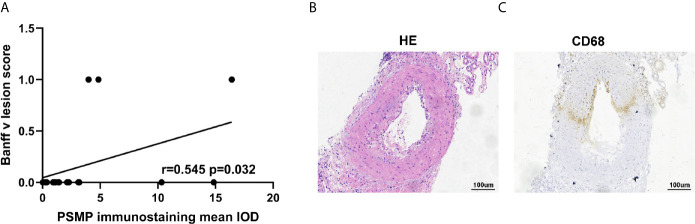
The relationship between PSMP expression and v-lesion in CAAMR patients. Correlations between PSMP expression and Banff v-lesion scores **(A)** were analyzed by Pearson’s correlation coefficient. The infiltration of CD68^+^ cells were shown in chronic intimal arteritis by immunohistochemistry staining **(B, C)**.

We analyzed the correlation between PSMP expression quantified by immunohistochemistry staining in renal biopsies and the Banff criteria scores in CAAMR patients. The results showed that PSMP expression was significantly associated with the v-lesions in CAAMR patients (p < 0.05, **Figure 6A**), but no significant correlation was identified with any other Banff lesion scores (**Figure S2A** **).** By analyzing the pathological data of patients with intimal arteritis (v) scores > 0 in CAAMR patients, the infiltration of CD68^+^ cells were found to be abundant in chronic intimal arteritis, suggesting that macrophage infiltration induced by the high expression of PSMP may promote the development of intimal arteritis (**Figures 6B, C**). We also found that in 3 cases with v-lesion (v > 0) in the CAAMR group, 2 cases experienced graft failure (**Table 2**). We also analyze whether PSMP levels were associated with graft function and the emergence of anti-HLA antibodies, which showed no significant correlations between PSMP expression and the eGFR or the median fluorescent intensity of anti-HLA II antibodies (**Figure S2B** **).**

**Table 2:** v lesion in CAAMR.

Characteristics	v>0 (n=3)	v=0 (n=14)	p value
CD68 number	259.33 ± 55.26	189.53 ± 111.37	0.33
Creatinine(mg/dl)	347 ± 78.87	216.87 ± 135.31	0.07
eGFR (ml/min/1.73 m^2^)	21.29 ± 26.034	36.23 ± 7.59	0.36
Graft loss-n (%)	2(66.67%)	1(7.14%)	0.07

eGFR, estimated glomerular filtration rate.

### PSMP Levels in the Serum and Urine Samples

**Figure 7:**
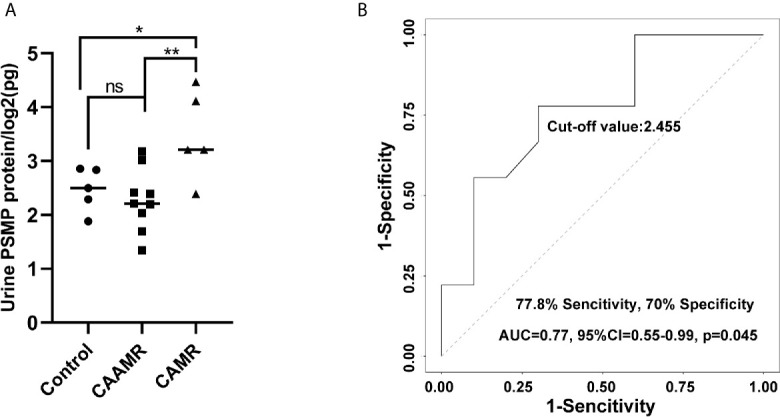
PSMP levels in the urine samples. Urine samples were collected and measured by flow cytometry-based cytokine bead assay **(A)**. ROC curves were used to evaluate the value of urine PSMP level in discriminating CAAMR patients from other patients **(B)**. *p ≤ 0.05, **p ≤ 0.01. ns, no significance.

We collected serum and urine samples of CAAMR, CAMR and Control patients, the PSMP expression levels were detected using the flow cytometry-based cytokine bead assay. However, The PSMP levels were lower in CAAMR groups compared with CAMR patients in the urine samples, (p < 0.05, **Figure 7A** **)**. Based on the ROC curve analysis, urine PSMP levels could distinguish CAAMR patients from other patients, with an area under the ROC curve (AUC) of 0.77 (95% confidence interval (0.55 to 0.99), a specificity of 70%, and a sensitivity of 77.8% (p < 0.05, **Figure 7B** **).** No significant differences in serum PSMP levels were observed among the three groups (**Figure S3A** **)** and could not be used as biomarkers to distinguish CAAMR patients from the other patients (**Figure S3B** **).**

## Discussion

In this study, we explored potential diagnostic and therapeutic biomarkers for chronic renal transplant rejection, include CAAMR and CAMR. Long-term graft failure caused by CAAMR has been a key challenge in renal transplantation (18). Chronic rejection needs pass through an early and active chronicity rejection phase (7) before the eventual develop to chronic graft injury. The diagnosis and treatment of early chronicity is important for blocking the progress of chronic graft injury. Although CAMR accompanied by significant microvascular inflammation was defined as CAAMR in the revised 2013 Banff classification (19), the diagnostic criteria for CAAMR have been continuously refined (20). Some specific cases that distinguish the two renal rejection types were not well known which lead to graft failure due to delayed treatment. We have shown that graft survival and function of CAAMR and CAMR patients were significantly reduced compared with those in the non-rejection group, suggesting the important effects of early active chronic development processes on graft function. Therefore, early diagnosis and treatment of CAAMR can avoid irreversible fibrosis and hold back the progress to CAMR. In our study, PSMP expression in graft biopsy tissues may be more meaningful for the diagnosis of CAAMR and distinguishing from CAMR. We have found that PSMP protein and mRNA levels both increased in CAAMR biopsy tissue, was a significant discriminatory factor that can be used to distinguish CAAMR patients from CAMR and non-rejection patients. However, there was no significant difference of PSMP protein expression in blood samples of CAAMR patients. We concluded that PSMP expression in graft biopsy tissues may be a distinguishing feature for CAAMR and should be considered an important histopathological diagnostic criterion in renal transplantation rejections. In addition, we observed that urine PSMP decreased in CAAMR patients and also can discriminated CAAMR patients from other patients. While the mechanism underlying PSMP production and metabolism is not yet clear, we will further explore this phenomenon in animal models.

According to our results, we believe that PSMP might be an important factor in the development of chronic antibody-mediated rejection. PSMP is a chemokine that recruits active monocytes-macrophages and promotes M1 polarization during the inflammatory response (17). Monocytes and macrophages infiltrate significantly in antibody-mediated rejection and can be serve as predictor of graft failure (21). In chronic rejection, macrophage infiltration plays a more important role than T cells and is positively correlated with poor graft prognosis (8, 22). We found that macrophages significantly infiltrated in CAAMR patients, PSMP expression was significantly associated with CD68^+^ macrophage accumulation in CAAMR graft biopsy tissues. M1 macrophages secrete pro-inflammatory cytokines that participate in the development of chronic rejection, whereas M2 macrophages secrete anti-inflammatory cytokines to promote tissue repair or graft fibrosis through the macrophage-to-myofibroblast transition (MMT) (23, 24). The M2 macrophages accumulation increased significantly 1–5 years after transplantation involved in the chronic injure progression (12), which may be associated with the fibrosis and decreased graft function (25, 26). We found CD163^+^ M2 cells increased in CAAMR but lower than that in CAMR patients, revealed that M2 macrophages might play a more prominent role in CAMR than in CAAMR. In addition, PSMP expression was associated with CD68^+^ macrophage infiltration but not with the accumulation of CD163^+^ M2 macrophages in CAAMR. Therefore, we believe that PSMP expression increase and promote M1 macrophage accumulation in early CAAMR, followed by M2 polarization and decreased PSMP expression in late CAAMR. However, we require more additional data and experiments to verify this hypothesis.

Intimal arteritis caused by vascular rejection has previously considered to be a feature of acute cellular rejection (1, 27). In the revised 2013 Banff criteria, vascular arteritis (v > 0) was included in the diagnostic criteria for active antibody-mediated rejection (19). Although isolated v-lesion has been associated with reduced recipient graft survival (28, 29), this has not been evaluated in CAAMR. In CAAMR patients, 3 of 17 cases presented intimal arteritis, of which 2 of 3 grafts were lost, indicating a significant relationship between v-lesion and graft loss in CAAMR. Although there was no significant significance which might due to small size of v> 0, we can still observe that patients with v > 0 has lower graft function and higher graft failure in CAAMR patients. Anyway, more samples remain necessary to verify this relationship between v-lesion and CAAMR. Although only 3 cases (v > 0) were identified, a significant correlation was found between the expression level of PSMP and v-lesion detection in CAAMR. In acute rejection, macrophages significantly infiltrated in the interstitium and arterial intima of vascularized grafts (30, 31), but little known about the macrophages and arterial intima in CAAMR. We found that intimal arteritis showed increased macrophage infiltration in CAAMR, which may be due to the recruitment effect of PSMP on macrophages. Macrophages may be a therapeutic target for improving the long-term outcome of grafts (32). It has been proved that inhibiting the accumulation of macrophages in the graft can effectively maintain the kidney graft function and prolong the survival of the graft (33). We found that PSMP promoted macrophage accumulation in grafts of CAAMR patients, may be serve as a therapeutic target for prevention macrophages infiltration and chronic graft failure in CAAMR.

In summary, our results revealed that PSMP was significantly increased in CAAMR patients but not in CAMR patients, and PSMP expression in graft biopsy tissues was a significant discriminative biomarker that distinguishes CAAMR from CAMR patients. A significant correlation was found between the expression of PSMP and CD68^+^ macrophage infiltration in CAAMR. Our data also revealed that v-lesions in CAAMR contributed to increased graft loss, and PSMP expression levels were significantly associated with v-lesion. These results indicated that PSMP played an important role in CAAMR development by recruiting macrophages to the renal transplant tissue and can be used as a discriminative histopathological diagnostic biomarker and therapeutic target for CAAMR.

## Data Availability Statement

The original contributions presented in the study are included in the article/**Supplementary Material**. Further inquiries can be directed to the corresponding author.

## Ethics Statement

The studies involving human participants were reviewed and approved by Ethics Committee of Tianjin First Central Hospital (2020N228KY). Written informed consent for this study was not required in accordance with the national legislation and the institutional requirements.

## Author Contributions

PZ: research performing and manuscript writing. HL: data acquisition and statistical analysis. MH: provided guidance for planning. ZW: collection and assembly of data. JZ: pathological diagnosis of the kidney biopsies. JT: technical supporting. XS: interpreted the data. YF: conception and design of the entire study. All authors contributed to the article and approved the submitted version.

## Funding

This study was supported by the National Natural Science Foundation of China (81970654).

## Conflict of Interest

The authors declare that the research was conducted in the absence of any commercial or financial relationships that could be construed as a potential conflict of interest.
