# Supplementary material for: PSMP Is Discriminative for Chronic Active Antibody-Mediated Rejection and Associate With Intimal Arteritis in Kidney Transplantation
Source: Front Immunol. 2021 Apr 9;12:661911. doi: 10.3389/fimmu.2021.661911 (PMC8062877; doi:10.3389/fimmu.2021.661911)
Supplement: Supplementary file 1 [file DataSheet_1.docx]

**Supplementary Figure 1**


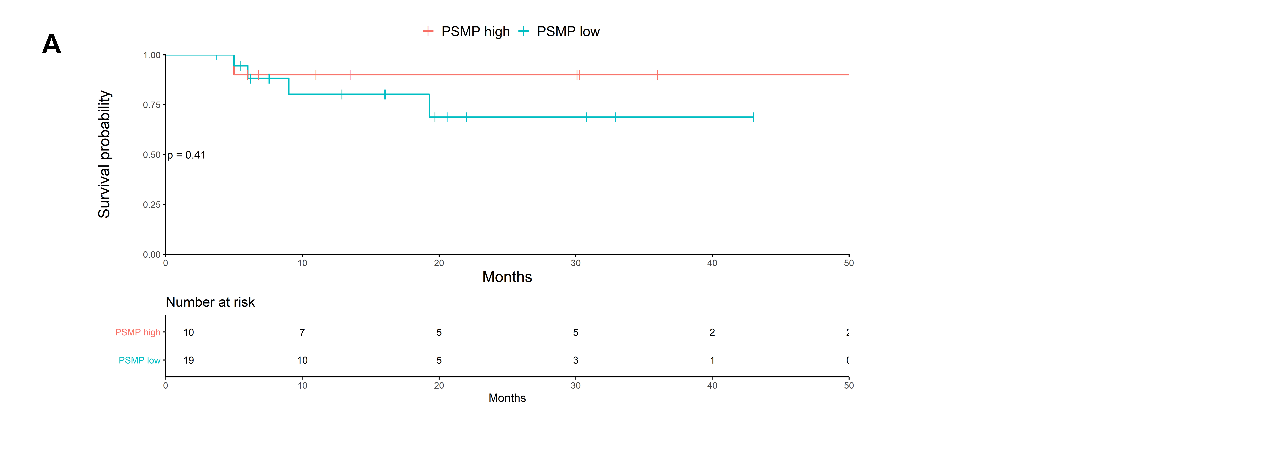
 Figure S1| Comparison of Survival rate between high and low expression of PSMP. Kaplan-Meier survival analysis was performed at the cut-off value of PSMP protein in biopsy to compare graft survival between high- and low-PSMP expression groups among CAAMR patients.

**Supplementary Figure 2**


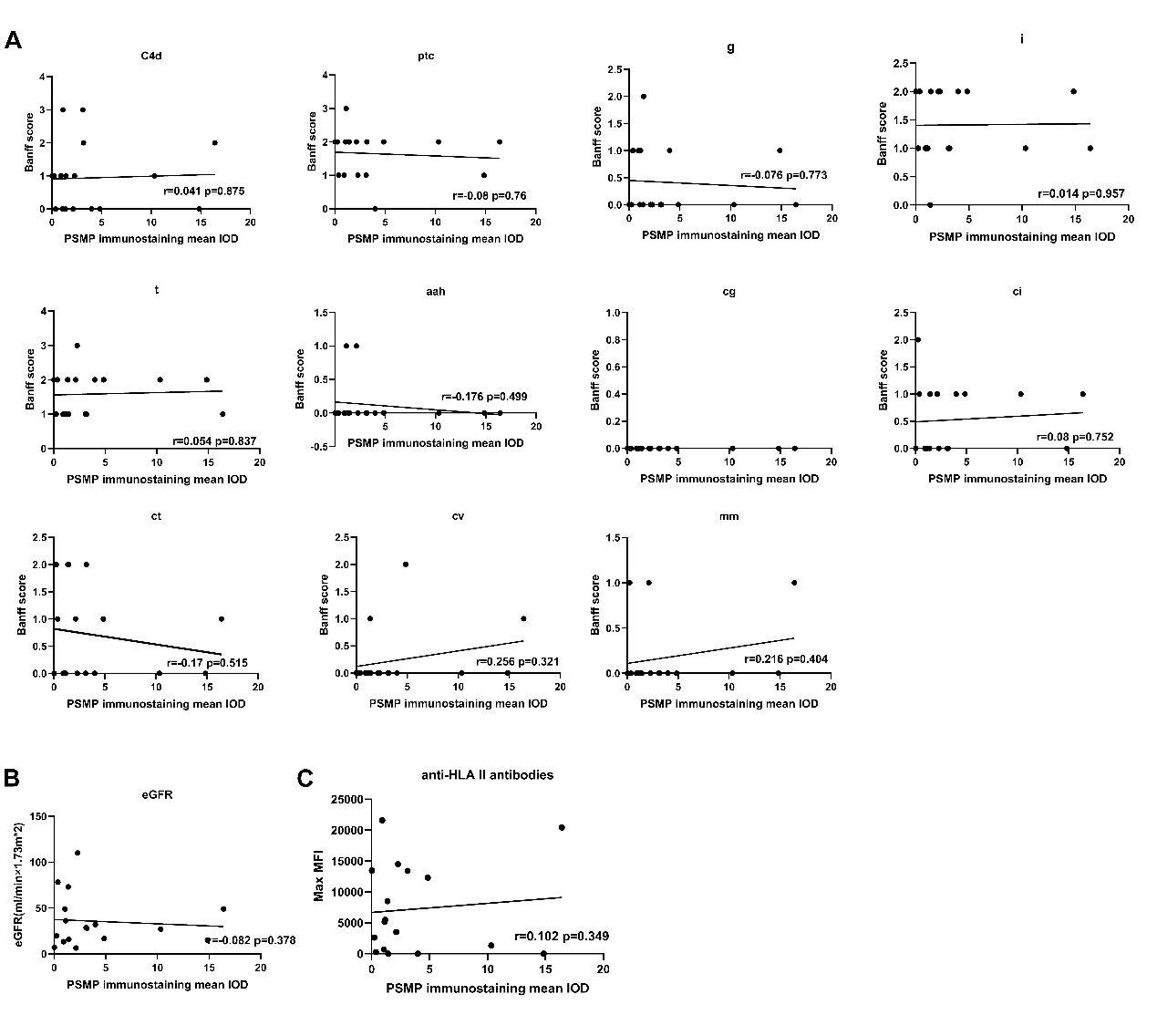


Figure S2| The relationship between PSMP expression and pathological grading and renal function in CAAMR patients. Correlations between Banff Lesion scores (C4d, ptc, g, i ,t, aah,cg, ci, ct, cv, mm) (A), eGFR (B) and anti-HLA II antibodies (C) were analyzed by Pearson’s correlation coefficient.

**Supplementary Figure 3**


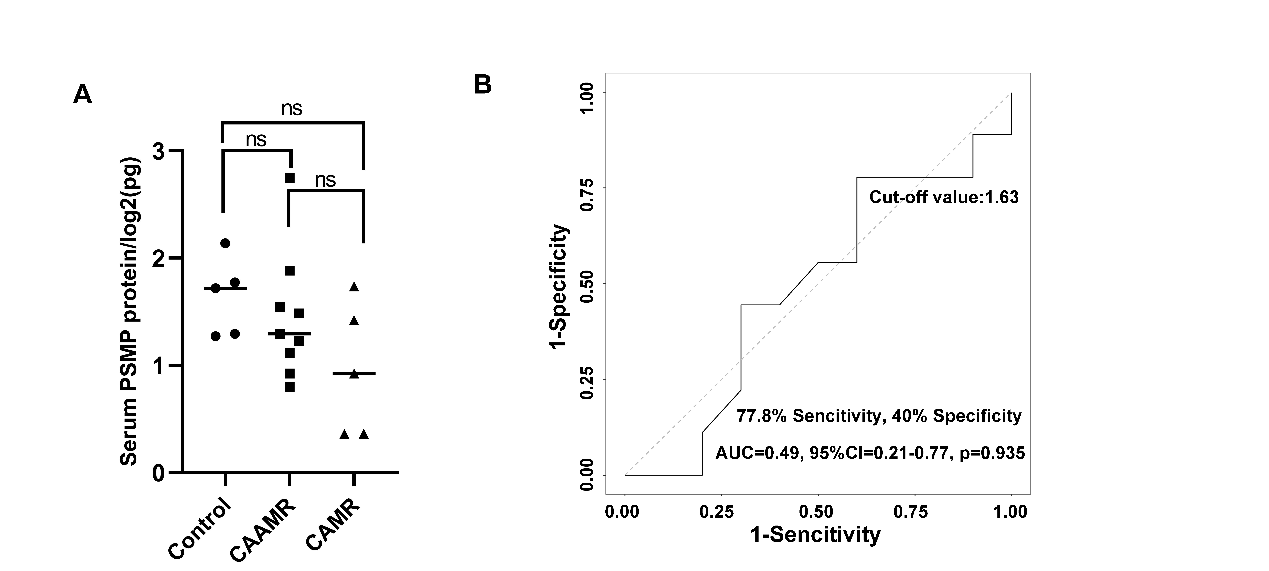


FigureS3 | PSMP levels in the serum samples. Serum samples were collected and measured by flow cytometry-based cytokine bead assay(A). ROC curves were used to evaluate the value of PSMP level in discriminating CAAMR patients from other patients(B). ns: no significance.
